# Airway Complications from an Esophageal Foreign Body

**DOI:** 10.1155/2016/3403952

**Published:** 2016-12-12

**Authors:** Ismael Garcia, Joseph Varon, Salim Surani

**Affiliations:** ^1^Facultad de Medicina Tampico, Universidad Autónoma de Tamaulipas, Tampico, TAMPS, Mexico; ^2^Dorrington Medical Associates, Houston, TX, USA; ^3^Foundation Surgical Hospital, Houston, TX, USA; ^4^The University of Texas Health Science Center at Houston, Houston, TX 77030, USA; ^5^Texas A&M University, Corpus Christi, TX 78413, USA

## Abstract

*Introduction*. Foreign body impaction (FBI) in the esophagus can be a serious condition, which can have a high mortality among children and adults, if appropriate diagnosis and treatment are not instituted urgently. 80–90% of all foreign bodies trapped in the esophagus usually pass spontaneously through the digestive tract, without any medical or surgical intervention. 10–20% of them will need an endoscopic intervention.* Case Report*. We hereby present a case of a large chicken piece foreign body impaction in the esophagus in a 25-year-old male with mental retardation. Patient developed hypoxemic respiratory failure requiring intubation. The removal required endoscopic intervention.* Conclusions*. Foreign bodies trapped in the upper gastrointestinal tract are a serious condition that can be fatal if they are not managed correctly. A correct diagnosis and treatment decrease the chances of complications. Endoscopic treatment remains the gold standard for extracting foreign body impaction.

## 1. Introduction

A foreign body impaction (FBI) in the esophagus can be a serious condition with high mortality rate among children and adults. A foreign body can be defined as the presence of any object, food, or material in the upper gastrointestinal tract, swallowed by accident or intentionally [1]. Children are more commonly affected by these conditions than adults. In the adult population, certain special conditions, such as mental retardation, psychiatric disorders, alcohol intake, and demented or edentulous patients, put them in a higher risk for developing an FBI [2].

Studies have shown that 80–90% of all foreign bodies trapped in the esophagus pass spontaneously whereas the remaining 10–20% of cases will require an endoscopic intervention to remove the FBI [3].

Radiological imaging of the neck and abdomen can allow the clinician to identify the radiopaque object and complications as esophageal perforation [4]. There are various ways to achieve removal of a FBI; these include nonendoscopic methods and endoscopic methods, which include flexible endoscopy versus rigid endoscopy. The rigid endoscopy is considered the gold standard for the treatment of FBI [5]. In cases when the airway of the patients seems compromised the use of a rigid endoscopy and intubation are the best treatment option [6, 7]. The choice by the clinician relies on the patient condition, the characteristics of the object, and the location, type, form, size of material, object or food that got impacted, and the anatomical portion of the esophagus which gets affected, and the duration of FBI episode [8].

We hereby present a case of a foreign body in the esophagus caused by a food bolus impaction with a piece of chicken in a 25-year-old male with mental retardation; patient developed hypoxemic complications which were resolved. This was managed endoscopically via using flexible video endoscope by Olympus.

## 2. Case Report

25-year-old gentlemen presented to the emergency department (ED) due to acute shortness of breath and bronchospasms after ingesting the chicken piece. Patient past medical history was significant for mental retardation, bipolar disorder, seizure disorder, and hypertension.

Patient was eating a chicken piece for meal, according to the witness he started to have a choking episode, Heimlich maneuver was performed, and a piece of the chicken was expelled. He started having severe respiratory distress after the incident and was transferred to the ED via ambulance. On arrival in ED, the patient was in significant respiratory distress. Patient was placed on oxygen supplementation with nonrebreather mask at 15 L/min. On auscultation stridor was heard in upper airway and rhonchi were heard in all lung fields. Patient blood pressure was 162/97 mmhg, heart rate was 103 beats/min, respiratory rate was 30/min, and pulse oximetry showed an oxygen saturation of 100%. Patient temperature was 99.3°F. An X-ray with lateral view of the neck was performed, showing no radiopaque foreign body within the pharyngeal or laryngeal region. Cervical spine X-ray to the level of C5 was within normal limits. Patient WBC count was 18.5 mm^3^. Electrolytes and electrocardiogram were within normal limits. Patient underwent an emergent bronchoscopy in the ED revealing no foreign body and normal airway. Patient continued to have respiratory distress and bronchospasms, which failed to improve postracemic epinephrine and steroid. Patient was intubated with excellent arterial blood gas (ABG) with no significant A-a gradient. Patient was also placed on empiric broad-spectrum antibiotic with piperacillin and tazobactam. In the following day, patient had an excellent oxygenation on arterial blood gas. Patient was extubated. Immediately after extubation patient went into severe respiratory distress and bronchospasm, requiring immediate reintubation.

**Figure 1:**
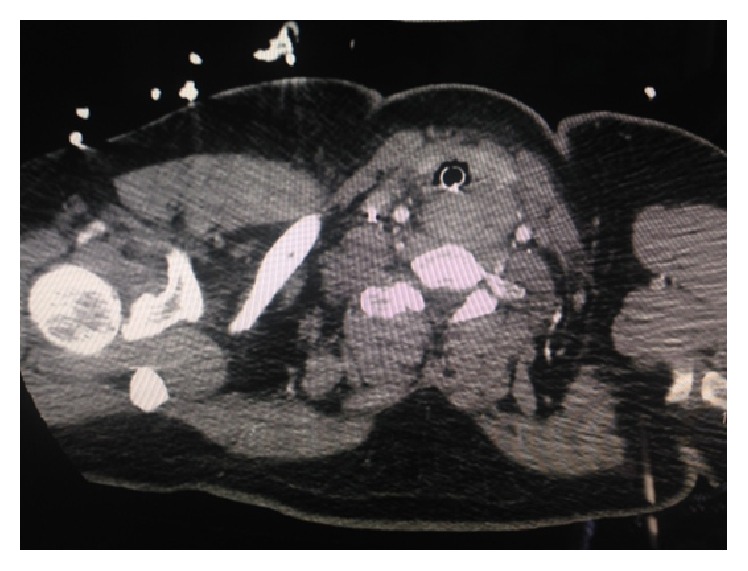
Computerized tomography of chest demonstrating foreign body impaction versus mass in esophagus.

CT scan of the chest was performed which showed a large soft tissue mass 8 × 6 cm posterior to the trachea, extending to the left of midline posterior to the left thyroid lobe (Figure 1). The mass displaces the trachea anteriorly and slightly to the right. Differential diagnosis included esophageal mass versus large left thyroid versus possible nonopaque foreign body. Other findings reported were posterior right upper lobe and bilateral lower lobe consolidations.

In the view of the patient history of possible foreign body ingestion, patient underwent esophagogastroduodenoscopy (EGD). The flexible video endoscope was inserted and passed without difficulty up to the upper esophageal sphincter, where a very large piece of chicken was identified occluding the proximal esophagus and causing significant pressure on the posterior tracheal wall. A tunnel in the middle of the chicken piece was made and grabbed in pieces with a snare and eventually removed as much as possible, weakening the center piece so the rest of the piece could pass easily into the stomach. The scope was advanced into the duodenum, which was in the normal limits without any acute findings.

Following the EGD, the patient was able to be successfully extubated. Patient initially received intravenous antibiotics, which was switched to oral antibiotics, and was discharged home on oral antibiotics in 3 days.

## 3. Discussion

The foreign body impaction (FBI) is considering an emergent situation. FBI is defined as the presence of any object, material, or food that gets trapped in the upper gastrointestinal tract, usually swallowed by accident or in some cases, intentionally. Some data reports that around 100,000 of FBI occur each year in the United States of America [9]. This event can lead to high morbidity and mortality [10]. It is estimated that between 1,500 and 1,600 patients die yearly due to FBI and esophageal perforation being the most dreaded complication [11].

We presented a case of a FBI in a 25-year-old adult. Although children's are the ones most commonly affected (specially between 6 and 72 months of age), [6, 10–12], our patient with history of mental retardation made him a high-risk person for FBI. In addition, other factors for adults include gastrointestinal alterations [9, 12, 13], psychiatric disorders, alcohol/drug intoxication, being edentulous elderly, baseline dementia, or altered mental status [8, 11].

Numerous objects and food can get impacted in the upper gastrointestinal tract [6, 10, 11, 14]. In our case a piece of chicken was swallowed. The literature reveals that in adults FBI with food occurs more frequently, especially meat products, fish, or chicken bones [15]. Among the pediatric population, coins and small batteries are the most common objects [6, 14].

Majority of the FBIs do not need any kind of intervention or treatment, data reports that around 80 and 90% of the FBIs will pass from the esophagus to the stomach without any intervention, the remaining 10–20% will need endoscopic intervention, and 1% of the FBIs cases will require surgical intervention [6, 8, 10, 14, 15]. In our case the need of endoscopic intervention was needed as it compromised the airway by extrinsic pressure on the membranous wall of the trachea, leading to the tracheal collapse. The clinical presentation as seen in our patient causing airway compromise is seen in 10% of the cases [16]. In some cases, patients with FBI may be asymptomatic, to be diagnosed later on during imaging studies or examination as an incidental finding. In other circumstances, patient presents with array of symptoms (see Table 1) [11, 14, 16].

In most cases making an accurate diagnosis is simple, as patient presents with the history or has been witnessed, and other times it can be complex, especially in case of very small children or adult with dementia or mental retardation that may not be able to provide adequate history. In our case, the information presented was provided by the family members of our patient and a choking episode with the chicken piece was witnessed. When ingestion of a foreign body is suspected, either by symptoms, when present, or by clinical history imaging studies with X-ray of neck, chest, and abdomen may help in diagnosis [17]. We performed an X-ray with lateral view of the neck and no radiopaque foreign body was seen in the pharyngeal or laryngeal region, as literature suggests [18, 19]. In the event of the negative X-ray and patient with a high index of suspicion for FBI, we performed a chest computerized tomography as our next step for diagnosis, which reported the presence of a mass posterior to the trachea, as mentioned above.

In any case of FBI the first line of treatment is to protect the airway [8]. Our patient presented with symptoms of respiratory distress and was intubated. The management used in our case was the flexible video endoscopic removal of the foreign body. The flexible endoscopy which is readily available and can be done at the bedside was the first choice. Though rigid scope can be used instead or in the cases when flexible scope fails to remove the FBI [14]. Minor complications have been reported in literature when endoscopic methods are performed [19]. The chicken piece in our patient was successfully removed with a flexible endoscope and patient then was successfully extubated and discharged home. Regardless of the methodology used after removal of the foreign body, follow-up imaging studies need to be done to ensure complete removal and rule out any complication.

FBI, though having a low complication rate, error in diagnosis or the delay in the management of these situations can lead to very critical and life treating situations. The most severe of them is perforation of the esophageal wall, which can lead to mediastinitis, abscess, fistula formation, empyema, sepsis, and death [11, 13, 15, 20–22]. Other life treating complications are airway compromise as occurred in our patient, which was treated initially by endotracheal intubation and later endoscopic removal of the FBI. Other complications from FBI are direct damage to the esophageal wall and migration of foreign body to trachea or mediastinum [6, 18].

## 4. Conclusions

Foreign bodies trapped in the upper gastrointestinal tract are a serious condition that can be fatal if not managed correctly. Accurate diagnosis and urgent treatment decrease the complications risk. Although majority of these events resolve spontaneously by themselves, some do require intervention. Endoscopic treatment remains as the standard for extracting foreign body impaction. Physicians need to perform adequate history and if unavailable or in doubt imaging studies need to be done to identify the FBI and site of impaction.

## Figures and Tables

**Table 1:** Clinical manifestation of foreign body impaction.

System	Symptoms
Gastrointestinal	(i) Abdominal pain (ii) Dysphagia (iii) Halitosis (iv) Hematemesis (v) Nausea (vi) Odynophagia (vii) Regurgitation (viii) Vomiting

Respiratory	(i) Cough (ii) Drooling (iii) Dyspnea (iv) Stridor (v) Wheezing
